# Evaluation of l-cell activity in the small intestine according to the extension of the biliopancreatic loop in patients undergoing Roux-en-Y gastric by-pass

**DOI:** 10.1016/j.clinsp.2024.100555

**Published:** 2025-01-27

**Authors:** Priscila Costa Estabile, Márcia Saldanha Kubrusly, Robson Kiyoshi Ishida, André Bubna Hirayama, Roberto de Cleva, Marco Aurelio Santo

**Affiliations:** Hospital das Clínicas da Faculdade de Medicina da Universidade de São Paulo (HCFMUSP), São Paulo, SP, Brasil

**Keywords:** L Cell, GLP-1, PYY, Biliopancreatic Loop, Gastric Bypass

## Abstract

**Background:**

Individuals with severe obesity and type 2 diabetes mellitus have reduced secretion of incretins by L cells. Studies suggest an increase in L cell activity according to the length of the Biliopancreatic Loop (BPL).

**Objective:**

Compare the effect of biliopancreatic loop extension on the number and expression of L cells in patients undergoing RYGB

**Methods:**

Subjects (*n* = 13) undergoing RYGB with a BPL of 100 cm (G1) or 200 cm (G2). Intestinal biopsies were done before (T1) and 6 months after (T2) RYGB in 3 segments: gastro-enteric anastomosis (A), entero-enteric anastomosis (B) and terminal ileum (C). Analyzes of intestinal biopsies by immunohistochemistry and qRT-PCR.

**Results:**

There was an increase (*p* < 0.0001) in L cells marked by PYY and GLP1 between T1 (17 ± 10.5) and T2 (23.5 ± 10.7) only at point C. There was no difference in L cells expression between groups G1 and G2 at points A (A1: 17.3 ± 2.9; A2: 19.9 ± 1.9; *p* = 0.09), B (B1: 13.7 ± 6.6; B2: 14.1 ± 4.9; *p* = 0.89) and C (C1: 13.2 ± 2.2; C2: 11.4 ± 3.4; *p* = 0.32) in PYY and GLP1 gene expression (A1: 20.8 ± 4, 1; A2: 23.7 ± 3.6; *p* = 0.2), B (B1:14.3 ± 7.9; B2: 22.7 ± 11.8; *p* = 0.1), (C1: 17±4.1; C2: 21.1 ± 4.8; *p* = 0.2).

**Conclusions:**

Both techniques lead to an increase in the number of active L cells.

## Highlights

• Question – Extension of the biliopancreatic loop increases l-cell activity after surgery bariatric?
• Findings – l-cell activity increased independent of extension of the biliopancreatic limb.
• Meaning – The additional beneficial effects of long BPL may be due to more rapid delivery of food and bile salts to more distal portions of the intestine.

## Introduction

Metabolic/bariatric surgery has been shown to be more effective than medical/lifestyle interventions in remission of Type 2 Diabetes (T2DM), including among individuals with class I obesity, for whom surgery is not widely used.^1^^,^^2^

Roux-en-y Gastric Bypass (RYGB) contributes to the improvement of the physiological and metabolic response due to the resumption of intestinal hormonal signaling, improvement of comorbidities, and weight loss on long-term follow-up.^3^^,^^4^

Enteroendocrine L cells are essential in the regulation of appetite and glucose homeostasis. They are distributed throughout the human gastrointestinal tract, but patients with severe obesity and Type 2 Diabetes (T2DM) had a reduced number of L cells and also alterations in the processing and release of gut-derived hormones.^5^^,^^6^

RYGB technique modifications were proposed to have a more impactful and lasting metabolic response after surgery. Some studies suggested that a long Biliopancreatic Limb (BPL) had superior effects in T2DM remission.^7^ These improvements may be, at least in part, the result of RYGB-induced rerouting of nutrients, which in turn is thought to alter the secretion of several gut-derived hormones.^7^^,^^8^

Different lengths of the intestinal loop could potentiate the physiological and biochemical response due to more intense intestinal hormonal signaling since nutrients that stimulate the gut-derived hormones could reach quickly more distal intestinal portions where L cells are located.^9^^,^^10^

The authors hypothesized that a long BPL allows for quick contact of food with a portion of the small intestine with a greater population of L cells, contributing to increased release of gastrointestinal hormones.

## Methods

A prospective study involving 13 patients (18‒65 years old) with a Body Mass Index (BMI) > 35 kg/m^2^ and T2DM undergoing RYGB between February 2020 and December 2022. All procedures were performed in accordance with the ethical standards of the institutional and national research committee and the 1964 Helsinki Declaration and its later amendments. This study was approved by the Human Research Ethics Committee (n° 65,854,317.8.0000.0068) and registered at Clinical Trials (NTC 05,446,414).

Patients were randomized (https://hcbredcap.com.br/) in a 1:1 ratio by computer model into two groups according to the length of the BPL: G1 (BPL = 100 cm; *n* = 6) and G2 (BPL = 200 cm; *n* = 7), both with an alimentary loop of 100 cm. Patients were evaluated before (T1) and 6-months (T2) after RYGB.

### Study protocol

#### Acquisition of samples

Intestinal biopsies were performed in 3 segments: gastroenteric anastomosis (a), enteroentero anastomosis (b) and terminal ileum (c). Preoperative biopsies were performed by colonoscopy and at surgery. Postoperative biopsies were performed 6 months after RYGB by colonoscopy and antegrade enteroscopy (Fig. 5).

The samples submitted to qRT-PCR analysis were stored in RNAlater™ solution (Qiagen) and maintained at a temperature of −80 °C, while samples for immunohistochemical analysis were placed in formol 10 % solution for 24 h and stored in 70 % ethanol at 4 °C until use. The assays were realized on intestinal mucosa samples for GLP-1 and PYY incretins produced by L cells.

#### qRT-PCR analysis

To perform qRT-PCR, 100 mg of RNA was extracted from the tissue that was processed using the Trizol extraction protocol. For cDNA transcription, the High-Capacity RNA-to-cDNA kit (50 reactions-Thermo Fisher) was used, following the manufacturer's instructions. For amplification, the authors used the following reaction conditions: 10.0 µL of TaqMan® Gene Expression Master Mix 2 ×, 1.0 µL of Taqman® Gene Expression Assay 20 ×, 5.0 µL RNase-DNase free water, and 4.0 µL of cDNA (1 to 100 ng) adding up to a total of 20 µL for each reaction. The following cycling parameters were used: 2 min at 50 °C, 10 min at 95 °C, followed by 40 cycles at 95 °C for 15 s and 60 °C for 1 min on the Applied Biosystems StepOnePlus™ thermal cycler.

#### Immunohistochemical analysis

Immunohistochemical analysis was performed on paraffin-embedded tissue, which was cut into sections for mounting slides. The histological sections were treated by serial washing to block endogenous peroxidase and activate the specific antigen for each antibody used. Histological sections were incubated with the primary antibodies (diluted in PBS) overnight at 4 °C, the primary antibody dilution was 1:1000.

### Statistical analysis

A non-parametric repeated measures ANOVA model was used. All tests had a bidirectional α of 0.05 and a Confidence Interval (CI) of 95 %. Descriptive statistics including mean, standard deviation, median, minima, and maxima were calculated to summarize continuous and quantitative variables.

## Results

### PYY

**Fig. 1:**
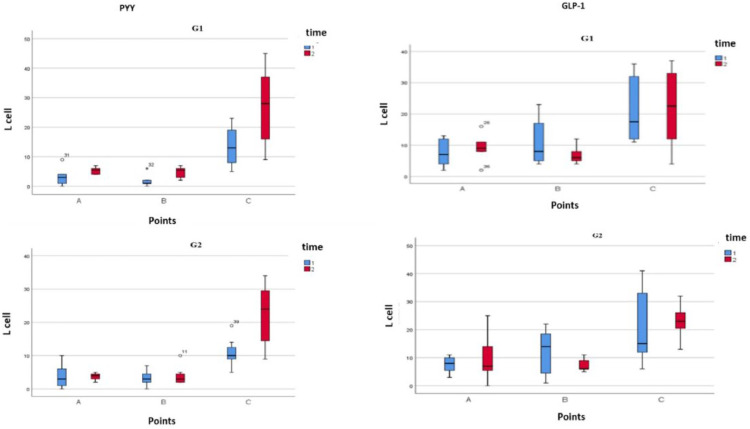
Boxplot of total L Cells at G1 and G2. Identified by anti-PYY(letf) and anti-GLP-1 (right) in T1 and T2.

Intestinal biopsies exhibit normal histology in all segments. There was an increase (*p* = 0.002) in the number of L cells (Fig. 1) immunostained by PYY in T2 (23.5 ± 10.7) in relation to T1 (17 ± 10.5).

**Fig. 2:**
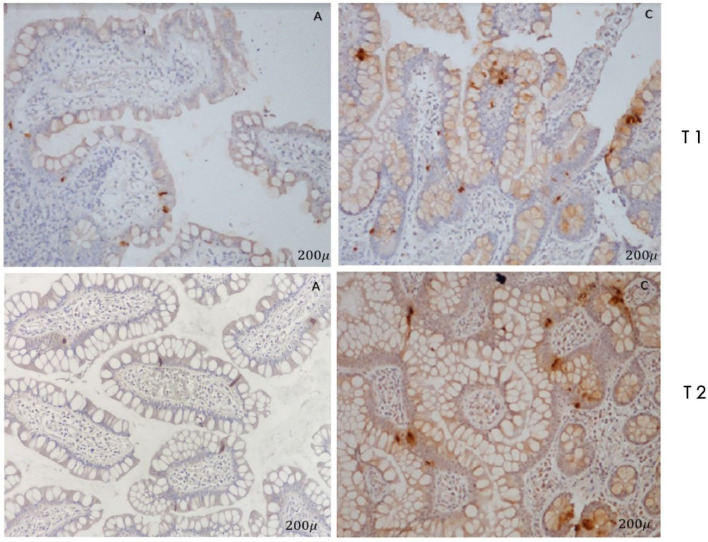
Immunostaining with polyclonal antibody PYY. Note the increase in the number of L cells in point C in relation relative to point A.

Fig. 1 represents the relative effect of RYGB on L cell number, with a linear and continuous increase being observed at point C in all patients, while points A and B did not change significantly between periods T1 and T2. There was a significant increase (*p* < 0.0001) in L cells number only in point C (Fig. 2). There was no difference between points A, B, and C between G1 and G2.

In T1 and T2 the numbers of L cells were maintained in G1 and G2 at points A and B. There was an increase in L cells (Table 1) only in point C in T1 (*p* = 0.002) and T2 (*p* = 0.001) in both groups.

**Table 1:** Descriptive analysis of L cells number according to extension of BPL at T1 and T2.

PYY	G1 (*n* = 6)		G2 (*n* = 7)	
Point	T1	T2	T1	T2
A	3.3 ± 3.1	4.5 ± 1.4	3.9 ± 4.4	3.6 ± 3.5
B	1.8 ± 2.1	4.4 ± 2.4	3.3 ± 2.4	2.6 ± 2.3
C	13.5 ± 6.7	24.5 ± 11.7	3.3 ± 2.4	12.2 ± 5.5

GLP-1	G1 (*n* = 6)		G2 (*n* = 7)	
Point	T1	T2	T1	T2
A	7.5 ± 4.5	9.7 ± 6.6	7.6 ± 3.0	7.5 ± 3.6
B	10.8 ± 7.8	7.2 ± 2.6	11.9 ± 8.5	11.4 ± 7.2
C	21 ± 10.5	22.5 ± 9.6	23.1 + 13.3	22.1 ± 11.7

G1, BPL of 100 cm; G2, BPL of 200 cm; T,1Preoperative; T2, 6-moths; A, Gastroenteric anastomosis; B, Entero-enteric anastomosis; C, Terminal ileum (C).

### GLP-1

The results of the total L cell count identified by GLP-1 are shown in Fig. 1. There was no significant difference between points A, B, and C between G1 and G2. There was a significant increase (*p* < 0.0001) only in point C.

**Fig. 3:**
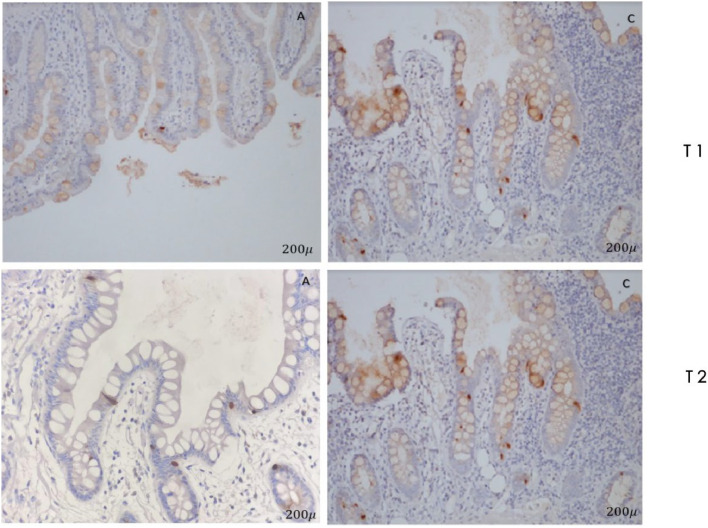
Immunostaining with polyclonal antibody GLP-1. Note the increase in the number of L cells in point C in relation relative to point A.

In immunostaining by anti-GLP-1, a significant increase (*p* = 0.009) of L cells in point C in relation to point A was observed (Fig. 3).

The number of l cells in T1 (9.5 ± 3.5) and T2 (8 ± 3.5) at points A and B was maintained in G1 and G2 with a significant increase (*p* = 0.002) at point C (Table 1).

In T1 and T2 the numbers of L cells were maintained in both groups (G1 and G2) at points A and B. There was an increase in the numbers of L cells only in point C in T1 (*p* = 0.002) and T2 (*p* = 0.003) (Table 1)

### mRNA PYY expression

**Fig. 4:**
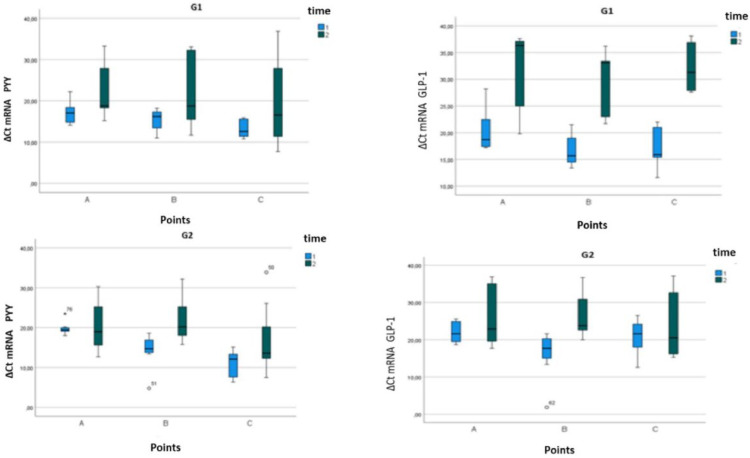
Boxplot of mRNA ΔCt value at G1 and G2. For PYY(left) and GLP-1(right) target genes in T1 and T2.

Expression of mRNA PYY was elevated after RYGB. There was no difference (Table 2) in PYY gene expression between G1 (17.3 ± 0.3) and G2 (16.6 ± 1) although there was a significant increase (*p* < 0.0001) at points A, B, and C after RYGB in both groups (Fig. 4).

**Table 2:** mRNA ΔCt value for G1 and G2 target genes in T1 and T2.

PYY	G1 (*n* = 6)		G2 (*n* = 7)	
Point	T1	T2	T1	T2
A	17.2 ± 2.8	22 ± 6.9	19.8 ± 1.7	20.5 ± 7.1
B	15.3 ± 2.7	21.6 ± 9.8	14.2 ± 4.5	22.1 ± 6.4
C	13.1 ± 2.2	19.5 ± 11.1	10.7 ± 3.5	17.1 ± 9.3

GLP-1	G1 (*n* = 6)		G2 (*n* = 7)	
Point	T1	T2	T1	T2
A	20.8 ± 4.6	31.1 ± 8.2	22 ± 2.8	26.2 ± 8.1
B	16.8 ± 3.3	29.4 ± 6.6	16.2 ± 6.3	26.4 ± 6.2
C	17.1 ± 4.2	32.3 ± 4.9	20.8 ± 4.7	23.9 ± 9.2

G1, BPL of 100 cm; G2, BPL of 200 cm; T1, Preoperative; T2, 6-moths; A, Gastroenteric anastomosis; B, Entero-enteric anastomosis; C, Terminal ileum (C).

**Fig. 5:**
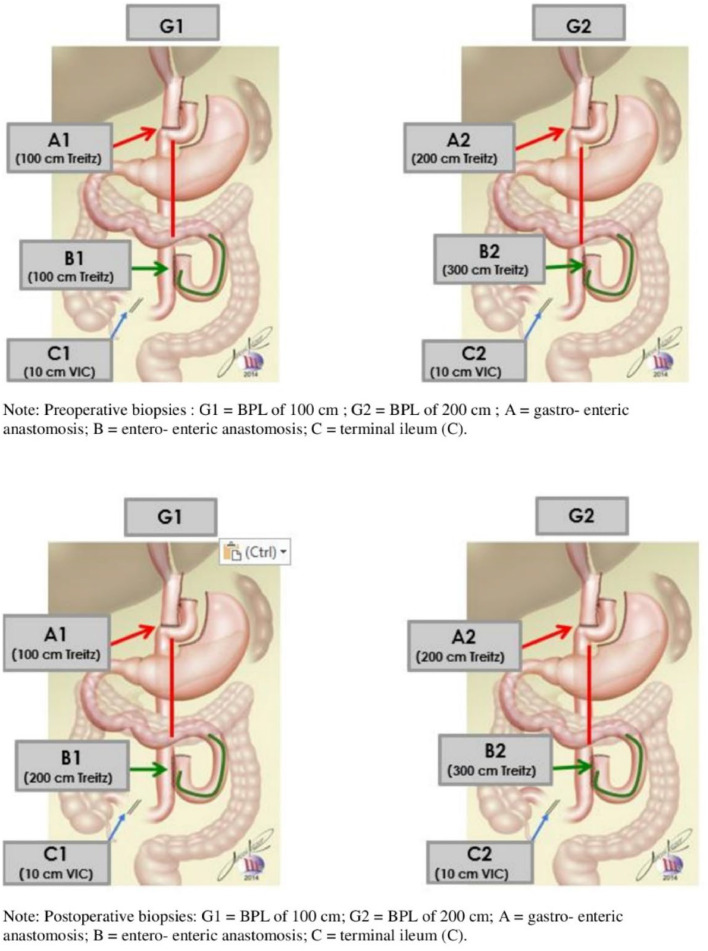
Preoperative biopsies: G1, BPL of 100 cm; G2, BPL of 200 cm; A, Gastro-enteric anastomosis; B, Entero-enteric anastomosis; C, Terminal ileum.

### mRNA GLP-1 expression

There was a significant increase (*p* = 0.016) in GLP-1 gene expression before (T1: 19.1 ± 4.49) and after (T2: 27.6 ± 7.74) RYBG. No significant difference was observed between groups G1 and G2 (Table 2). There was an increase in points A, B, and C, (*p* < 0.00010) after RYGB in both groups (Fig. 4).

## Discussion

This study analyzes the effects of a long or standard BPL after RYGB in L cell activity. The present study also provides the transcriptional activity and gene expression of L cells in the mucosa of the small intestine. The authors only compared changes in response to L cell activity and gene expression before and after the patient underwent RYGB.

Some studies suggest a hybrid mechanism in RYGB: an improvement of the enteric hormone secretion associated with small intestinal malabsorption.^11^^,^^12^ A previous study demonstrated that patients with obesity and T2DM undergoing RYGB had an early improvement in insulin resistance along with an increase in GLP-1 days after surgery.^13^

Ingested food normally stimulates the release of satiety-stimulating gut hormones and insulin, such as GLP-1 and PYY. This response is attenuated in patients with obesity and insulin resistance but is rapidly restored after RYGB.^13^ The authors hypothesize that metabolic changes observed after RYGB could be accentuated by a long BPL due to overstimulation of L cell activity.^13^

There was an increase in the total number of GLP-1 cells months to years after RYGB as a result of overgrowth of the jejunum mucosa. In the ileum and colon, significant morphological changes occurred, including an increase in the villi height with hyperplasia of the mucous neck cells concomitant with increased density of GLP-1 positive cells.^14^

Considerable reserves of GLP-1 and PYY are stored in intestinal endocrine L cells^14^ RYGB with a longer BPL results in a distinct postprandial hormone profile with increased GLP-1 and neurotensin responses that may be beneficial to the metabolic outcomes of surgery than the classic procedure.^15^, ^16^, ^17^

An increase in density of active L cells results in improved peptide signaling, response and function of the neuroendocrine system. A greater number of L cells is observed in more distal segments of the small intestine in the late postoperative period of RYGB.^18^

Nevertheless, another study demonstrates that a longer BPL in RYGB does not affect GLP-1 secretion and suggested that the characteristic increase in response to GLP-1 after RYGB may not depend only on the delivery of nutrients to more distal intestinal segments.^19^

The L cell determines the secretory responses to different stimuli according to their anatomical location and cell maturation.^15^ L cells are fully renewed within 7 days, a short time in comparison to other endocrine cells.^15^

In this study, the authors found no difference in L cell activity between a long BPL (G2) and standard BPL (G1). The increase in l-cell density was observed after RYGB in both groups. Then, the beneficial effects observed on glucose homeostasis may be due to more rapid delivery of food and bile salts to more distal portions in long BPL.^20^

In the present study, biopsy collection was performed at 20-cm gastro-enteric and enterro-enteric anastomosis, following recommendations in the literature.^15^^,^^21^ The size of the long BPL loop was chosen because it is the place with the largest population of the L cells.^19^

The authors observed a significant difference in L cells number only in point C. Some studies have already shown that the L cell population is greater in the most distal portion of the small intestine.^21^ Even so, there was no difference between groups G1 and G2 either in RNA expression, Then, both techniques determine improved incretin response and contribute to remission of T2DM.^22^

In RYGB, shortened route of the biliopancreatic limb and the common limb determines earlier contact and more active bile acid reabsorption with the ileum.^23^

TGR5 (GPR131) and G protein-coupled receptor 1 are other receptors involved in maintaining glucose homeostasis. After RYGB there is an increase in serum and luminal bile acids that activated the G-protein coupled bile acid Receptor (TGR5) on L cells, resulting in the release of GLP1. Bile acids also induce the synthesis and secretion of FGF19 in ileal cells, improving insulin sensitivity and regulating glucose metabolism.^23^^,^^24^

## Conclusion

There was a significant increase in density and expression of L cells after RYGB, without difference between standard or long BP loops.

## Authors' contributions

Priscila Costa Estabile: Preparation of study, data collection, research execution and drafting of the text. Márcia Saldanha Kubrusly: Research execution and drafting of the text. Robson Kiyoshi Ishida: Data collection. André Budna Hirayama: Pathological analysis. Roberto de Cleva: Drafting of the text. Marco Aurélio Santo: Research execution and drafting of the text.

## Conflicts of interest

The authors declare no conflicts of interest.
